# Breast Cancer Treatment Disparities in a Rural Setting: Conserving Surgery Versus Mastectomy

**DOI:** 10.3390/jcm14093264

**Published:** 2025-05-07

**Authors:** Benjamin C. Kensing, Lutfi A. Barghuthi, Marvin Heck, Carly R. Wadle, Rebecca J. Swindall, Alan D. Cook, Hishaam N. Ismael

**Affiliations:** 1 Department of Surgery, University of Texas Health Science Center Tyler, Tyler, TX 75708, USA; benjamin.kensing@uttyler.edu (B.C.K.); lutfi.barghuthi@uttyler.edu (L.A.B.); marvin.heck@uttyler.edu (M.H.); alancook@uttyler.edu (A.D.C.); hishaam.ismael@uttyler.edu (H.N.I.); 2 School of Medicine, University of Texas Health Science Center Tyler, Tyler, TX 75708, USA; carly.wadle@uttyler.edu

**Keywords:** breast-conserving therapy, lumpectomy, patient preference mastectomy, rural healthcare, breast cancer, disparities

## Abstract

**Background/Objectives:** Randomized controlled trials demonstrate comparable survival among early-stage breast cancer patients undergoing breast-conserving therapy or patient preference mastectomy. Many factors affect the choice of treatment like the availability of radiation centers, socioeconomic status, and insurance status. This study aimed to identify the determinants of surgical breast cancer treatments in a rural community. **Methods:** Retrospective data were obtained from the medical records of breast cancer patients between 2015 and 2022 at a single rural healthcare system. Demographics, barriers to care, support services offered, pre-treatment services, and the type and stage of cancer were analyzed to identify trends among patients who received breast-conserving therapy and mastectomy. **Results:** Among the 162 patients who underwent a mastectomy, 16.1% chose this procedure based on patient preference. The patient preference mastectomy group was younger with a median age of 58 years compared to 65 years in the breast conservation group. Additionally, they were 2.7 times more likely to choose a mastectomy when reporting no financial support. When receiving lymphedema management or psychosocial services, they were also more likely to be in the patient preference mastectomy group, 58.3% versus 5.2% and 100% versus 83.5%, respectively. Genetic screening, however, was more common among the breast conservation therapy group (61.9% vs. 26.9%). **Conclusions:** Our findings indicate an increase in the utilization of breast conservation therapy in a rural healthcare system. These patients were generally older, had financial support, and received genetic screening. Having a multidisciplinary approach to treating breast cancer contributes to our ability to pursue breast-conserving therapy measures in rural communities.

## 1. Introduction

Breast cancer was historically treated with radical resection. The first radical mastectomy was developed and performed by William Halsted in New York in 1882. Radical mastectomy involves removing the entire breast, all axillary lymph nodes, and the pectoral muscles. It is a morbid procedure that left women disfigured and often unnecessarily so in the context of early and small tumors. Eventually, surgeons realized that removing the pectoral muscles did not improve survival; therefore, the modified radical mastectomy was developed, which spared the pectoral muscles. Around the same time in the mid-20th century, surgeons began treating early-stage tumors with partial mastectomies (i.e., lumpectomies) and incorporating radiation therapy as an adjunct treatment. Despite early studies showing no difference in survival, mastectomy remained the gold standard of breast cancer treatment for much of the 20th century [1]. In the 1970s, the National Surgical Adjuvant Breast and Bowel Project (NSABP) began investing in breast-conserving therapy, the treatment of breast cancer with partial mastectomy combined with adjuvant radiation. The NSABP B-06 trial was a cornerstone randomized control trial that demonstrated no survival difference in women with early-stage breast cancer who underwent mastectomy or lumpectomy followed by radiation [2]. This led to the adoption of breast-conserving therapy as an accepted treatment modality for breast cancer.

Since then, numerous randomized controlled trials have shown that survival rates for patients with early-stage breast cancer are comparable whether they undergo breast-conserving therapy or patient preference mastectomy [3,4]. As a result, the rates of breast-conserving therapy have increased nationwide over the last few decades. However, despite the evidence supporting the effectiveness of breast-conserving therapies, a significant portion of women continue to undergo mastectomies. Many of these women are guided toward mastectomies due to absolute or relative contraindications to breast-conserving therapy, as outlined in clinical guidelines. Yet, there remains a subset of patients who opt for a mastectomy in the absence of any contraindication, raising important questions about the underlying factors influencing surgical decision making in breast cancer treatment.

The reasons behind choosing patient preference mastectomy over breast-conserving therapy are multifactorial and, in some instances, unclear. Several factors have been proposed that may affect a woman’s choice of surgical treatment such as age, race, and socioeconomic status [5,6,7,8,9]. Additionally, living in rural geographic regions introduce structural barriers such as limited access to radiation therapy facilities, a necessary component of breast-conserving therapy, which may disproportionately affect breast cancer patients [5,6,7,8,9]. Studies have documented that breast cancer patients living in rural areas face unique challenges, including presenting with more advanced disease at diagnosis and decreased survival when compared to urban breast cancer patients [10,11].

The purpose of this study is to identify and characterize disparities in breast cancer diagnosis and treatment, with a particular focus on rural populations. Cancer disparities encompass a range of differences in various metrics such as incidence, prevalence, mortality, morbidity, survival rates, screening practices, staging at diagnosis, and delays in treatment [12]. Understanding these disparities is critical to developing targeted interventions that improve access to care and improve outcomes for underserved rural populations.

By examining the determinates of surgical breast cancer treatment among breast cancer patients residing in rural communities, we aim to identify key factors influencing treatment selection and the barriers preventing access to breast-conserving therapy. The findings from this study may help inform policy changes and healthcare interventions that promote patient-centered care and reduce disparities in breast cancer treatment and outcomes among patients residing in rural regions.

## 2. Materials and Methods

We conducted a retrospective single-institution study of breast cancer patients 18 and older between January 2015 and October 2022 in a rural East Texas population. East Texas consists of 35 counties existing within the Texas Health and Human Services Public Health District 4/5 North including 1.5 million people with a population density of 48 people per square mile compared to 112 across the rest of the state [13,14]. Upon diagnosis with breast cancer, the standard of care involves the following: (1) treatment recommendation from the surgeon, (2) consultation with a patient care coordinator, and (3) continued education given by the patient care coordinator. The patient care coordinator is responsible for identifying barriers to care including access to medical resources, transportation challenges, the perceived adequacy of social or support services, the impact of comorbidities, ability to comply with the treatment plan, fixed income status, and citizenship. Based on individual needs, support services are discussed and tailored for each patient. The patient care coordinator of this institution prospectively maintained a dataset documenting the reported barriers and support services provided. This study was granted a waiver of Institutional Review Board review (18-009).

**Figure 1:**
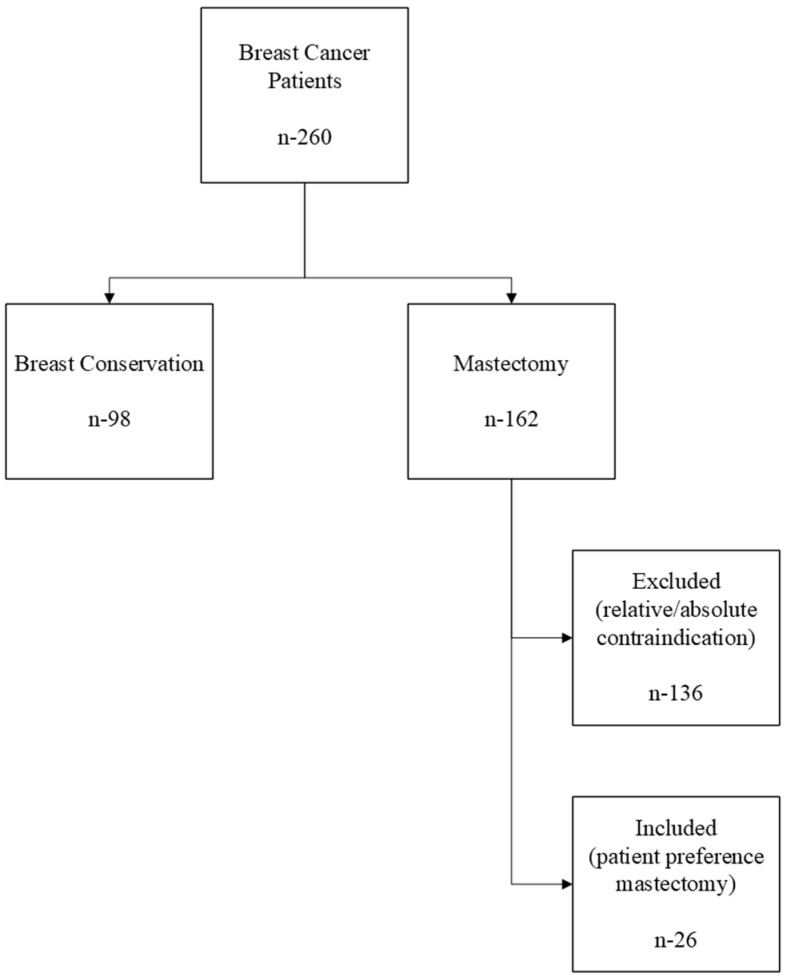
Breast conservation therapy and patient preference mastectomy flow chart.

Relevant clinical measures were retrospectively retrieved from electronic medical records, including, age, sex, marital status, insurance status, race/ethnicity, whether cancer was recurring, timeliness of care, plastic surgery referral, pre-treatment consultation, type of exam, screenings conducted, and diagnosis. Exclusion criteria for this study included if a patient underwent mastectomy due to an absolute or relative contraindication for breast-conserving therapy (Figure 1) based on National Comprehensive Cancer Network practice guidelines.

Demographic variables, barriers to care, available support services, pre-treatment services, cancer type, and cancer stage were analyzed to identify trends among patients who received breast-conserving therapy and those who, despite qualifying for breast-conserving therapy, opted for a mastectomy. Patient characteristics, barriers to care, and support services offered are presented as numbers and percentages, means and standard deviations (SDs), or medians and interquartile ranges (IQRs). Associations between categorical variables were assessed using the Pearson chi-square test or Fisher’s exact test. Differences between continuous variables were examined with an ANOVA and the Kruskal–Wallis test by ranks, as appropriate. Crude odds ratio and 95% confidence intervals were reported using logistic regression analysis. The results of this study were considered statistically significant with a *p*-value < 0.05. Statistical analysis was conducted using Stata16.1 (Stata, Inc., College Station, TX, USA).

## 3. Results

Of the 124 patients included in this study, 98 (79%) underwent breast-conserving therapy, and 26 (21%) opted for a mastectomy even though they were eligible for breast conservation. The reasons for choosing mastectomy for their cancer care treatment are listed in Table 1. The patient preference mastectomy group was younger than the breast-conserving therapy group, with a mean (SD) age of 58 ± 14 compared to 65 ± 11 years (*p* = 0.02), with decreasing odds of choosing a mastectomy as the patient aged (OR: 0.95; 95% CI: 0.92 to 0.99). Marital status and race/ethnicity were similar between groups. Most were married (54.6%) and White or non-Hispanic (75.8%); we also found no differences between groups. We did find that insurance status varied slightly between surgery groups, where 19.2% of the patient preference mastectomy group was uninsured compared to 9.2% of the breast-conserving therapy group; however, these findings did not reach statistical significance, likely due to the small sample size. Further, women who chose to have a mastectomy were also more likely to report that they lacked financial support, 34.6% compared to 16.3% (OR: 2.71; 95% CI: 1.03 to 7.15), *p* = 0.04. Additionally, 23.1% of the patient preference mastectomy group reported that they also lacked social support compared to 11.2% of the breast-conserving therapy group. See Table 2.

Differences were observed between surgery groups and the utilization of support services. Lymphedema management was significantly more common in the patient preference mastectomy group compared to the breast-conserving therapy group, 88.3% vs. 5.2%, respectively (OR: 25.8; 95% CI: 7.76 to 86.55), *p* < 0.001. Psychosocial services were received by all from the patient preference mastectomy group, compared to 83.5% of the breast-conserving therapy group, *p* = 0.02. No differences were found among other support services offered between treatment groups. See Table 3.

Most patients were diagnosed with invasive ductal carcinoma (77.4%); once again, there were no differences found between treatment groups. Pre-treatment consultations were received by 82.8% of all patients, with a 7% difference between groups, 88.5% compared to 81.1% of breast-conserving therapy patients. Recurring cancer was rare among this patient population, 4.1% of the breast-conserving therapy group and none in the patient preference mastectomy group. There was a 10% difference between groups diagnosed with stage 1 cancer, 60.2% of the breast-conserving therapy group compared to 50% of the patient preference mastectomy group. A higher proportion of women with stage 2 disease was found among the patient preference mastectomy group, 34.6% compared to 16.1% of the breast-conserving therapy group. Neoadjuvant treatment as the first form of treatment was rare (1.7%). Among patients who received genetic evaluation, 61.9% of the breast-conserving therapy group received a screening compared to 26.9% of the patient preference mastectomy group. The opposite is true concerning genetic testing, 69.2% of the patient preference mastectomy group compared to 38% of the breast-conserving therapy group, *p* = 0.002.

## 4. Discussion

We describe factors that may influence a breast cancer patient’s decision to choose to have a mastectomy even though they qualify for breast-conserving therapy. While the majority of the patients chose breast-conserving therapy, a significant portion still chose to receive a mastectomy despite being eligible for breast-conserving therapy. Younger age, financial barriers, and differences in the support services of lymphedema management and psychosocial services seem to play a role in their decision making. Additionally, the stage of cancer at diagnosis and type of genetic evaluation also appeared to influence this choice of surgery. We propose that both personal and clinical factors contribute to the patient treatment decision making process.

A multidisciplinary approach to treating breast cancer is essential, especially in rural regions where healthcare access may be limited. For example, having a patient care coordinator who counsels and educates patients can enhance the decision to choose breast-conserving therapy by ensuring patients are educated about what options are available to them. Several large national database studies have been conducted that compare treatment modalities in early-stage breast cancer. In 1992, Nattinger et al. reported that on average, 12.1% of women pursued breast-conserving therapy. Within a subset of women in the east south central US, only 5.9% of women underwent breast-conserving therapy [9]. In 2009, Smith et al. reported that 59% of patients underwent breast-conserving therapy [15]. More recently in 2015, Lautner et al. found, on average, that 60.1% of women received breast-conserving therapy and 52% opted for breast-conserving therapy within a subset of women in the southern United States [16]. Our findings confirm the increasing utilization of breast-conserving therapy, particularly in the southern United States, with nearly 80% of women who chose breast-conserving therapy when given the option.

The analysis of characteristics between the two treatment groups revealed some notable trends. Women who were younger opted for a patient preference mastectomy with a downward trend as the patient ages. Early studies comparing the use of BCT by age demonstrated conflicting results. Samet et al. evaluated the Surveillance, Epidemiology, and End Results (SEER) program data from 1983 to 1986 and demonstrated variation in BCT by age in different regions of the United States, generally favoring BCT in older patients [17]. Conversely, a more extensive analysis of the SEER program registries from 1983 to 1995 showed that BCT was favored in younger patients [18]. This variability can largely be attributed to rapidly changing guidelines surrounding the adoption of BCT during the late 20th century. Modern studies support the findings of our study and demonstrate that younger women are increasingly more likely to undergo mastectomy [19]. The decision to pursue mastectomy at a young age has been attributed to the fear of recurrence [20]. Furthermore, advancement in reconstructive and plastic surgery has provided favorable cosmetic outcomes which can be preferable to those of lumpectomy and breast radiation.

Another interesting finding was the higher likelihood of financial challenges amongst women choosing to undergo a patient preference mastectomy. Breast-conserving therapy has the added burden of postoperative radiation therapy which adds additional cost to treatment. These patients may also have increased barriers with the need to travel for this postoperative care and take more time off work. Women with fewer financial resources may naturally trend towards a mastectomy as a more cost-effective treatment modality. There was no significant difference in insurance status among women undergoing mastectomy or BCT in our study; however, there were meaningful differences between groups. The patient preference mastectomy group skewed towards being uninsured, 19.2% compared to 9.2%. Other studies have demonstrated a difference in treatment choice based on insurance status, particularly when comparing privately insured patients to Medicaid patients. Churilla et al. report rates of mastectomy of 55% for Medicaid and 52% for uninsured patients compared to 48% for privately insured patients [21]. Addressing existing financial disparities including the lack of insurance or financial support could help circumnavigate this barrier to provide equal access to breast-conserving therapy.

Support services also differed between surgery groups. Additional expected outcomes were related to lymphedema management and psychosocial services. Patients treated with mastectomy have a higher risk of developing lymphedema; therefore, a higher proportion will benefit from lymphedema management social services. Psychosocial services were utilized by all women undergoing mastectomy. The removal of a breast significantly impacts a woman’s body image and can manifest as fear, hopelessness, and depression [22]. It is critical to make these services readily available to women undergoing treatment for breast cancer.

While cancer type and stage were not statistically different between treatment groups, we did identify some trends. We found that a higher proportion of patient preference mastectomy patients were diagnosed with stage 2 cancer, compared to the breast-conserving therapy group. Prior retrospective reviews have also demonstrated trends towards mastectomy in stage 2 compared to stage 1 breast cancer [18]. This suggests that a woman with more advanced cancer may believe that a mastectomy is a more aggressive and final form of treatment, even when breast-conserving therapy is a viable option.

Genetic evaluation type was also found to influence the choice of surgery type. Screening was more common among the breast-conserving therapy group than the patient preference mastectomy group. However, a more detailed evaluation involving testing was more often performed in the patient preference mastectomy group. Further studies are needed to explore how genetic evaluation and counseling may impact patient care decision making.

Our findings highlight the need for specific interventions according to unique patient situations. Healthcare systems can aim to reduce financial burdens and increase social support by expanding financial assistance programs and improving access to support services. A focus on patient education can ensure that all women, regardless of socioeconomic status or regions within which they live, can make educated decisions based on the treatment options available to them. By identifying and understanding the key factors that influence treatment decisions, providers can finetune patient discussions to sooth concerns and correct misconceptions. Working towards unifying the responsibilities of breast cancer care coordinators and patient navigators may further improve decision making and access to breast conservation therapy in a rural setting.

This study has certain limitations. It is subject to biases due to its retrospective study design, including incomplete documentation and inability to control for confounding variables. Further, the small sample size limits statistical power to detect associations and may affect the generalizability of the findings to broader populations. The interpretation of this study’s findings should take into consideration these limitations.

On the other hand, this study also has notable strengths. Conducted at a single institution, it utilized a prospectively managed database, allowing for an increased depth of data, allowing us to exclude women with relative and absolute contraindications to breast-conserving therapy, limiting selection bias. Unlike some large database studies that rely on disease stage to assess eligibility for breast-conserving therapy, we incorporated National Comprehensive Cancer Network guidelines related to surgical recommendations to refine the analysis of treatment trends.

## 5. Conclusions

Breast-conserving therapy was the primary treatment modality among breast cancer patients in East Texas. Factors related to age and financial circumstances influenced treatment choice. Younger women and those with fewer financial resources were more likely to opt for mastectomy, even when clinically eligible for breast conservation. Patients who chose mastectomy also reported more psychosocial support, possibly reflecting the emotional and physical challenges they face following breast removal. The disparities identified among the patient preference mastectomy group highlight key areas for improving patient care, ultimately aimed at improving outcomes in a rural setting. Future research should focus on enhancing shared decision making and addressing psychological factors that influence treatment choices to optimize breast cancer care among underserved rural populations.

## Figures and Tables

**Table 1:** Reasons for mastectomy among 162 patients.

	N (%)
Widespread disease	59 (36.4)
Diffuse or microcalcifications	18 (11.1)
Radiation therapy during pregnancy	1 (0.6)
ATM mutation	0 (0)
Prior radiation therapy	12 (7.4)
Positive margins	15 (9.3)
Tumor >5 cm	41 (25.3)
Tumor location	21 (13.0)
Genetic predisposition	7 (4.3)
Connective tissue disease	2 (1.2)
Stage 3–4 disease	56 (34.6)
Patient preference	26 (16.1)

**Table 2:** Characteristics of 124 patients who had breast-conserving therapy and patient preference mastectomy.

	Total	Breast-Conserving Therapy 98 (79)	Patient Preference Mastectomy 26 (21)	*p*-Value
Age at diagnosis, mean (sd)	64 (12)	65 (11)	58 (14)	0.02
Died, n (%)	2 (1.6)	2 (2)	0 (0)	0.62
Marital status, n (%)				0.33
Single	20 (16.5)	15 (15.6)	5 (20)	
Married	66 (54.6)	52 (54.2)	14 (56)	
Divorced	8 (6.6)	5 (5.2)	3 (12)	
Widowed	27 (22.3)	24 (25)	3 (12)	
Race/ethnicity, n (%)				-
White	94 (75.8)	74 (75.5)	20 (76.9)	
Black	16 (12.9)	13 (13.3)	3 (11.5)	
Hispanic/Latino	13 (10.5)	10 (10.2)	2 (11.5)	
Asian	1 (0.8)	1 (1)	0 (0)	
Insurance status, n (%)				0.32
Uninsured	14 (11.3)	9 (9.2)	5 (19.2)	
Partially insured	19 (15.3)	16 (16.3)	3 (11.5)	
Insured	91 (73.4)	73 (74.5)	18 (69.2)	
Barriers, n (%)				
No barriers	47 (37.9)	36 (36.7)	11 (42.3)	0.60
No insurance	14 (11.3)	9 (9.2)	5 (19.2)	0.15
Inadequate insurance	23 (18.6)	19 (19.4)	4 (15.4)	0.44
Lack of medical resources	17 (13.7)	13 (13.3)	4 (15.4)	0.78
No transportation	3 (2.4)	2 (2)	1 (3.9)	0.51
No support services	22 (17.7)	16 (16.3)	6 (23.1)	0.42
Comorbidities	34 (27.4)	29 (29.6)	5 (19.2)	0.29
Compliance	8 (6.5)	6 (6.1)	2 (7.7)	0.53
No social support	17 (13.7)	11 (11.2)	6 (23.1)	0.12
No financial support	25 (20.2)	16 (16.3)	9 (34.6)	0.04
Fixed income	16 (12.9)	15 (15.3)	1 (3.9)	0.12
Non-US citizen	2 (1.6)	1 (1.0)	1 (3.9)	0.38

**Table 3:** Characteristics of 124 patients’ perioperative care and diagnosis patterns who had breast-conserving therapy and patient preference mastectomy.

	Total	Breast-Conserving Therapy 98 (79)	Patient Preference Mastectomy 26 (21)	*p*-Value
Support services, n (%)				
Lymphedema management	19 (15.7)	5 (5.2)	14 (58.3)	<0.001
Psychosocial services	105 (86.8)	81 (83.5)	24 (100)	0.02
Nutritional counseling	3 (2.5)	3 (3.1)	0 (0)	0.51
Palliative care	91 (75.2)	74 (76.3)	17 (70.8)	0.58
Transportation services	2 (1.7)	1 (1.0)	1 (4.2)	0.36
Financial services	31 (25.6)	24 (24.7)	7 (29.2)	0.66
Community health worker	77 (63.6)	60 (61.9)	17 (70.8)	0.41
American Cancer Society	115 (95)	91 (93.8)	24 (100)	0.21
Licensed social worker	30 (24.8)	23 (23.7)	7 (29.2)	0.58
Type of cancer, n (%)				-
Invasive ductal carcinoma	96 (77.4)	75 (76.5)	21 (80.8)	
Invasive lobular carcinoma	7 (5.7)	6 (6.1)	1 (3.9)	
Mixed/epithelial carcinoma	1 (0.8)	1 (1)	0 (0)	
Pre-treatment consult, n (%)	96 (82.8)	73 (81.1)	23 (88.5)	0.29
Recurring, n (%)	4 (3.2))	4 (4.1)	0 (0)	0.39
Stage of cancer				0.33
Stage 0	22 (18.5)	18 (19.4)	4 (15.4)	
Stage 1	69 (58)	56 (60.2)	13 (50)	
Stage 2	24 (20.2)	15 (16.1)	9 (34.6)	
Stage 3	2 (1.7)	2 (2.2)	0 (0)	
Stage 4	2 (1.7)	2 (2.2)	0 (0)	
Neoadjuvant treatment	2 (1.7)	1 (1.1)	1 (3.9)	0.51
Genetic evaluation				0.002
Not performed	1 (0.9)	0 (0)	1 (3.9)	
Screening	64 (54.2)	57 (61.9)	7 (26.9)	
Testing	53 (44.9)	35 (38)	18 (69.2)	

## Data Availability

The dataset presented in this article is not readily available because it is derived from a single-institution database and must ensure patient confidentiality. Requests to access the dataset should be directed to the corresponding author.

## Abbreviations

The following abbreviations are used in this manuscript:

SD	Standard deviation
IQR	Interquartile range
